# Supplementary material for: Hepatitis C virus exploits cyclophilin A to evade PKR
Source: eLife. 2020 Jun 16;9:e52237. doi: 10.7554/eLife.52237 (PMC7297535; doi:10.7554/eLife.52237)
Supplement: Supplementary file 1. [file elife-52237-supp1.docx]

**Supplementary File 1**

***Synthesis of novel CypI***

CsA-Prtc1 synthesis

**General Method A (metathesis)**

To a solution of Cyclosporin A (72 mg, 0.06 mmol) in DCM (2 mL) was added the olefin (0.072 mmol) and Hoveyda-Grubbs 2^nd^ generation catalyst (6 mg, 0.01 mmol, 17mol%). The reaction was stirred in the microwave at 90^o^C for 30 minutes and then allowed to cool. Triethylamine was added to the mixture and then stirred overnight with excess P(CH_2_OH)_3_ to coordinate the ruthenium catalyst. This was then washed away with brine and water before the mixture was passed through a Stratospheres PL Thiol MP SPE cartridge (polymer Lab, Varian Inc) to remove any remaining catalyst. The crude product was purified by chromatography (as detailed) to give the product*.*

**(2*S*,4*R*)-1-((*S*)-2-(hept-6-enamido)-3,3-dimethylbutanoyl)-4-hydroxy-N-((*S*)-1-(4-(4-methylthiazol-5-yl)phenyl)ethyl)pyrrolidine-2-carboxamide (JW4-7)**

To a solution of E3 ligase ligand 1 (0.072 g, 0.169 mmol) in MeCN were added 6-heptenoic acid (25 µL, 0.186 mmol), HATU (0.071 g, 0.186 mmol) and N,N-diisopropylethylamine (59 µL, 0.338 mmol) and this mixture was stirred at room temperature overnight. The mixture was then purified by column chromatography with 30-50% MeOH in DCM, and then re-purified with 25-35% MeOH in DCM.

The product was isolated as a pale yellow solid (46 mg, 49% yield).

^1^H NMR (700MHz, CDCl_3_) δ 8.81 (s, C=N, 1H), 3.39 (s, NMe, 3H), 8.20 (d, NH, 1H), 7.62 (d, NH, 1H), 7.29 (d, 2H), 7.19 (d, 2H), 5.59 (m, alkene, 3H), 4.95 (s, OH, 1H), 4.82 (q, 1H), 4.79 (q, 1H), 4.33 (d, 1H), 4.24 (t, 1H), 4.04 (sext, 1H), 2.32 (t, 3H), 2.26 (s, 3H), 2.07 (m, 1H), 2.01 (t, 1H), 1.93 (m, 1H), 1.83 (m, 2H), 1.60 (m, 1H), 1.30 (m, 2H), 1.15 (m, 2H), 0.75 (s, 9H).

^13^C NMR (600 MHz, CDCl_3_) δ 177.44, 176.01, 174.97, 156.85, 153.10, 150.01, 136.48, 135.03, 74.14, 63.88, 61.73, 61.57, 60.25, 53.94, 53.06, 43.04, 40.51, 40.01, 38.20, 33.16, 33.04, 31.76, 30.24, 29.35, 27.71, 21.29.

LCMS (ESI, *m/z*): [MH]^+^ calcd. for [C_30_H_42_N_4_O_4_S+H]^+^, 555.3005; found 555.3005.

**[Gly-(1*S*,2*R*,*E*)-10-amido (E3 ligase ligand)-1-hydroxy-2-methyloct-4-ene]^1^ CsA (CsA-Prtc1, JW4-10)**

**Using Method A**

The crude product was purified by flash silica chromatography 0-15% MeOH in DCM, then repurified with 5-9% MeOH in DCM to give **CsA-Prtc1**, **JW4-10** as an off-white solid (41 mg, 22% yield).

^1^H NMR (600 MHz, CDCl_3_) δ 3.48 (s, NMe, 3H), 3.39 (s, NMe, 3H), 3.22 (s, NMe, 3H), 3.11 (s, NMe, 3H), 3.09 (s, NMe, 3H), 2.69 (s, NMe, 3H), 2.68 (s, NMe, 3H).

^13^C NMR (600 MHz, CDCl_3_) δ 174.04, 173.79, 173.58, 173.51, 171.66, 171.30, 171.26, 170.53, 170.47, 170.22, 170.17.

HRMS (*m/z*): [MH]^+^ calcd. for C_89_H_147_N_15_O_16_S, 1715.0944; found 1715.0952.

JW115 synthesis

**1-(pent-4-en-1-yl)-1*H*-imidazole**

To a solution of imidazole (1.702 g, 25 mmol) in THF was added portionwise NaH (60% in mineral oil, 600 mg, 25 mmol). The resulting mixture was refluxed for an hour before cooling to room temperature and the addition of 5-bromo-pent-1-ene (3.25 ml, 27.5 mmol). The mixture was then refluxed for 3 hours, allowed to cool and diluted with diethyl ether. The organic extracts were combined, washed with brine, dried over MgSO_4_ and concentrated under reduced pressure. Product was purified with column chromatography 0-20% MeOH in DCM, followed by 9-12% MeOH in DCM.

The product was isolated as a transparent oil (648.5 mg, 68%).

^1^H NMR (600 MHz, CDCl_3_) δ 7.55 (s, 1H), 7.08 (s, 1H), 6.91 (s, 1H), 5.68-5.82 (m, 1H), 4.97-5.10 (m, 2H), 3.95 (t, *J*= 3.95 Hz, 3H), 2.01-2.11 (m, 2H), 1.85-1.92 (m, 2H).

^13^C NMR (600 MHz, CDCl_3_) δ 137.16, 136.77, 129.39, 118.85, 116.24, 46.25, 30.46, 30.08.

HRMS (*m/z*): [MH]^+^ calcd. for [C_8_H_12_N+H]^+^,137.1079; found 137.1079.

**[Gly-(1*S*,2*R*,*E*)-8-(1*H*-imidazole-1-yl)-1-hydroxy-2-methyloct-4-ene]^1^ CsA (JW115)**

**Using Method A**

The crude product was purified by flash silica chromatography 0-10% MeOH in DCM, and re-purified in 5-9% MeOH in DCM to give **JW115** as an off-white powder (11 mg, 10%).

^1^H NMR (600 MHz, CDCl_3_) δ 3.48 (s, NMe, 3H), 3.39 (s, NMe, 3H), 3.21 (s, NMe, 3H), 3.12 (s, NMe, 3H), 3.11 (s, NMe, 3H), 2.68 (s, NMe, 3H), 2.66 (s, NMe, 3H).

^13^C NMR (600 MHz, CDCl_3_) δ 173.92, 173.79, 173.55, 173.48, 171.67, 171.32, 171.23, 170.49, 170.44, 170.19, 170.16.

MS (*m/z*): [MH]^+^ calcd. for C_67_H_117_N_13_O_12_, 1296.73; found 1296.85.
