# Supplementary material for: Hepatitis C virus exploits cyclophilin A to evade PKR
Source: eLife. 2020 Jun 16;9:e52237. doi: 10.7554/eLife.52237 (PMC7297535; doi:10.7554/eLife.52237)
Supplement: Supplementary file 2. [file elife-52237-supp2.doc]

**Supplementary File 2**

Table 1. Oligo sequences

| **RNAi (pSIREN)** | | |
| --- | --- | --- |
| shCypA | Fwd | GATCCCCGGGTTCCTGCTTTCACAGATTCAAGAGATCTGTGAAAGCAGGAACCCTTTTTGGAAA |
|  | Rev | AGCTTTTCCAAAAAGGGTTCCTGCTTTCACAGATCTCTTGAATCTGTGAAAGCAGGAACCCGGG |
| shCypB | Fwd | GATCCGCCGGGTTCTTCATCACGACAGTCAACTCGAGTTGACTGTCGTGATGAAGAACTTTTTG |
|  | Rev | AATTCCAAAAAGTTCTTCATCACGACAGTCAACTCGAGTTGACTGTCGTGATGAAGAACCCGG |
| **CRISPR (lentiCRISPR v2)** | | |
| MAVS KO | Fwd | CACCGCAGGGAACCGGGACACCCTC |
|  | Rev | AAACGAGGGTGTCCCGGTTCCCTGC |
| PKR KO | Fwd | CACCGTAATACATACCGTCAGAAGC |
|  | Rev | AAACGCTTCTGACGGTATGTATTAC |
| IRF1 KO | Fwd | CACCGACAAGGATGCCTGTTTGTTC |
|  | Rev | AAACGAACAAACAGGCATCCTTGTC |
| **qRT-PCR** | | |
| ANKRD | Fwd | AATGTTGCCACCACTCTTCC |
|  | Rev | TGCAGCTGTGCATTCTTTTC |
| CCL2 | Fwd | CAGCCAGATGCAATCAATGCC |
|  | Rev | TGGAATCCTGAACCCACTTCT |
| CXCL2 | Fwd | GGGCAGAAAGCTTGTCTCAA |
|  | Rev | GCTTCCTCCTTCCTTCTGGT |
| CXCL10 | Fwd | TGGCATTCAAGGAGTACCTC |
|  | Rev | TTGTAGCAATGATCTCAACACG |
| GAPDH | Fwd | GGGAAACTGTGGCGTGAT |
|  | Rev | GGAGGAGTGGGTGTCGCTGTT |
| IFIT2 | Fwd | CAGCTGAGAATTGCACTGCAA |
|  | Rev | CGTAGGCTGCTCTCCAAGGA |
| IFN-β | Fwd | AGGACAGGATGAACTTTGAC |
|  | Rev | TGATAGACATTAGCCAGGAG |
| MX1 | Fwd | ATCCTGGGATTTTGGGGCTT |
|  | Rev | CCGCTTGTCGCTGGTGTCG |
| TNFα | Fwd | AGCCTCTTCTCCTTCCTGATCGTG |
|  | Rev | GGCTGATTAGAGAGAGGTCCCTGG |
| **Site-directed mutagenesis** | | |
| D316E/Y317N | Fwd | GGCACGGCCTGAGAACAACCCGCCGC |
|  | Rev | CAAGCCGGTAAGGCCCGTG |
| MAVS C508R | Fwd | GGCCTGTGGCGTGGCACCTCCCTCTCC |
|  | Rev | GGAGAGGGAGGTGCCACGCCACAGGCC |
| **Cloning** | | |
| pcDNA-MAVS | Fwd | GAGGTACCATGCCGTTTGCTGAAG |
|  | Rev | CAGGTCTAGACTAGTGCAGACGCC |
| **Sequencing** | | |
| NS5A DII | Fwd | GACTTTCTCGAGCCAATGG |
| U6 | Fwd | GAGGGCCTATTTCCCATGATT |
| CMV | Fwd | CGCAAATGGGCGGTAGGCGTG |
| BGH | Fwd | TAGAAGGCACAGTCGAGG |
