# Supplementary material for: Hepatitis C virus exploits cyclophilin A to evade PKR
Source: eLife. 2020 Jun 16;9:e52237. doi: 10.7554/eLife.52237 (PMC7297535; doi:10.7554/eLife.52237)
Supplement: Supplementary file 3. [file elife-52237-supp3.docx]

| **Key Resources Table** | | | | |
| --- | --- | --- | --- | --- |
| **Reagent type (species) or resource** | **Designation** | **Source or reference** | **Identifiers** | **Additional information** |
| gene (*Homo sapiens*) | PPIA (CYPA) |  | NCBI Gene:5478 |  |
| gene (*Homo sapiens*) | PPIB (CYPB) |  | NCBI Gene:5479 |  |
| gene (*Homo sapiens*) | PPIF (CYPD) |  | NCBI Gene:10105 |  |
| gene (*Homo sapiens*) | EIF2AK2 (PKR) |  | NCBI Gene:5610 |  |
| gene (*Homo sapiens*) | MAVS |  | NCBI Gene:57506 |  |
| gene (*Homo sapiens*) | DDX58 (RIG-I) |  | NCBI Gene:23586 |  |
| gene (*Homo sapiens*) | IFIH1 (MDA5) |  | NCBI Gene:64135 |  |
| gene (*Homo sapiens*) | IRF1 |  | NCBI Gene:3659 |  |
| strain, strain background (Hepatitis C Virus) | HCVcc | Apath LLC | mJ6/JFHRluc2  (APV24) |  |
| strain, strain background (*Escherichia coli*) | HB101 | Promega | L2015 | Chemically competent *E. coli* |
| cell line (*Homo sapiens*) | Huh7 | Dr. Yoshiharu Matsuura; originally from Japanese Collection of Research Bioresources Cell Bank | JCRB0403, RRID:CVCL_0336 |  |
| cell line (*Homo sapiens*) | Huh7.5 | Apath LLC  Blight et al., 2002 | APC166, RRID:CVCL_7927 |  |
| cell line (*Homo sapiens*) | Huh7.5-CTRL | Heit et al., 2002 |  |  |
| cell line (*Homo sapiens*) | Huh7.5-RIG-I | Heit et al., 2002 |  |  |
| cell line (*Homo sapiens*) | Huh7.5-Mda5 | Heit et al., 2002 |  |  |
| cell line (*Homo sapiens*) | Huh7.5-RIG-I/Mda5 | Heit et al., 2002 |  |  |
| cell line (*Homo sapiens*) | Huh7-Lunet | Friebe et al., 2005 | RRID:CVCL_U459 |  |
| cell line (*Homo sapiens*) | HEK293T | ATCC | CRL-3216, RRID:CVCL_0063 |  |
| transfected construct (human) | HCV SGR | Schaller et al., 2007 | pFKI389Luc/NS3-3’_dg_JFH | Electroporated into human hepatoma cell line |
| transfected construct (human) | HCV SGR-GND | Schaller et al., 2007 | pFKI389Luc/NS3-3’_dg_JFH_GND | Electroporated into human hepatoma cell line |
| transfected construct (human) | HCV SGR-DEYN | This paper | pFKI389Luc/NS3-3’_dg_JFH_DEYN | NS5A D316E/Y317N mutant HCV SGR. Electroporated into human hepatoma cell line, generated in Prof. Greg Towers’ lab (UCL) |
| transfected construct (human) | HCV protein expression construct | Romero-Brey et al., 2012 | pTM-NS3-5B | Transfected into human hepatoma cells |
| transfected construct (human) | p8.91 | Zufferey et al., 1997 |  | Lentiviral packaging plasmid |
| transfected construct (human) | pMDG | Naldini et al., 1996 |  | VSV-G envelope expressing construct |
| transfected construct (human) | pHIV-SIREN | Schaller et al., 2011 |  | Lentiviral transfer plasmid |
| transfected construct (human) | pLentiCRISPRv2 | Prof. Feng Zhang | RRID:Addgene_52961 | Lentiviral  transfer plasmid for Cas9-mediated KO |
| transfected construct (human) | pHIV-SIREN-shCypA | This paper |  | Lentiviral transfer plasmid encoding shRNA for CypA knockdown, generated in Prof. Greg Towers’ lab (UCL) |
| transfected construct (human) | pHIV-SIREN-shCypB | This paper |  | Lentiviral transfer plasmid encoding shRNA for CypB knockdown, generated in Prof. Greg Towers’ lab (UCL) |
| transfected construct (human) | pLentiCRISPRv2-sgPKR | This paper |  | Lentiviral transfer plasmid for PKR KO, generated in Prof. Greg Towers’ lab (UCL) |
| transfected construct (human) | pLentiCRISPRv2-sgMAVS | This paper |  | Lentiviral transfer plasmid for MAVS KO, generated in Prof. Greg Towers’ lab (UCL) |
| transfected construct (human) | pLentiCRISPRv2-sgIRF1 | This paper |  | Lentiviral transfer plasmid for IRF1 KO, generated in Prof. Greg Towers’ lab (UCL) |
| transfected construct (human) | pSCRPSY-EIF2AK | Feng et al. 2018 |  | Lentiviral transfer plasmid for PKR expression |
| transfected construct (human) | pcDNA3.1-MAVS-WT | This paper |  | Plasmid for expression of WT MAVS, generated in Prof. Greg Towers’ lab (UCL) |
| transfected construct (human) | pcDNA3.1-MAVS-C508R | This paper |  | Plasmid for expression of C508R MAVS, generated in Prof. Greg Towers’ lab (UCL) |
| antibody | Anti-NS5A (mouse monoclonal) | Dr. Joe Grove (UCL)  (Lindenbach et al., 2005) |  | IF (1:1000) |
| antibody | Anti-β-actin (mouse monoclonal) | Abcam | Cat# AB8226,  RRID:AB_306371 | WB (1:10000) |
| antibody | Anti-β-actin (rabbit polyclonal) | Abcam | Cat# AB8227, RRID:AB_2305186 | WB (1:1000) |
| antibody | Anti-CypA  (rabbit polyclonal) | Enzo | Cat# BML-SA296-0100, RRID:AB_2051206 | WB (1:5000) |
| antibody | Anti-CypB (rabbit polyclonal) | Abcam | Cat# AB16045, RRID:AB_443295 | WB (1:1400) |
| antibody | Anti-CypD (mouse monoclonal) | Abcam | Cat# AB110324, RRID:AB_10864110 | WB (1 μg/mL) |
| antibody | Anti-RIG-I (rabbit monoclonal) | Cell Signaling Technology | Cat# 3743, RRID:AB_2269233 | WB (1:1000) |
| antibody | Anti-MAVS (mouse monoclonal) | Santa Cruz Biotechnology | Cat# sc166583, RRID:AB_2012300 | WB (1:500) |
| antibody | Anti-PKR (rabbit monoclonal) | Abcam | Cat# AB32052, RRID:AB_2293421 | WB (1:1000) |
| antibody | Anti-phospho-PKR T446 (rabbit monoclonal) | Abcam | Cat# AB32036, RRID:AB_777310 | WB (1:1000) |
| antibody | Anti-IFNAR2 (mouse monoclonal) | Pbl Assay Science | Cat# 21385-1, RRID:AB_354167 | 2 μg/mL |
| antibody | IgG2A control antibody (mouse) | R&D Systems | Cat# 4460-MG-100, RRID:AB_884569 | 2 μg/mL |
| commercial assay or kit | Steady-Glo Luciferase Assay System | Promega | Cat# E2510 |  |
| commercial assay or kit | MEGAScript T7 Transcription Kit | Ambion, Life Technologies | Cat# AM1334 |  |
| commercial assay or kit | Neon Transfection System Kit | Life Technologies | Cat# MPK10025 |  |
| commercial assay or kit | Amaxa Nucleofecter System – Kit T | Lonza | Cat # VVCA-1002 |  |
| commercial assay or kit | TransIT LT1 Transfection Reagent | Mirus Bio LLC | Cat# MIR 2304 |  |
| commercial assay or kit | Fugene-6 Transfection Reagent | Promega | Cat# E2691 |  |
| commercial assay or kit | Q5 Site-Directed Mutagenesis | New England Biolabs | Cat# E0554S |  |
| commercial assay or kit | QuikChange II Site-Directed Mutagenesis | Agilent | Cat# 200523 |  |
| commercial assay or kit | Araldite 502/ Embed 812 kit | Electron Microscopy Sciences | Cat# 13940 |  |
| commercial assay or kit | RNeasy Mini Kit | Qiagen | Cat# 74106 |  |
| commercial assay or kit | SuperScript III Reverse Transcriptase | Invitrogen | Cat# 18080093 |  |
| commercial assay or kit | FastSYBR Green master mix | Applied Biosciences | Cat# 4385610 |  |
| commercial assay or kit | AlamarBlue Viability Assay | ThermoScientific | Cat# DAL1025 |  |
| chemical compound, drug | C16 | Sigma-Aldrich | Cat# I9785 |  |
| chemical compound, drug | Ruxolitinib | Cell Guidance Systems | Cat# SM87 |  |
| chemical compound, drug | Daclatasvir | Insight Biotechnology | Cat# D101505 |  |
| chemical compound, drug | Telaprevir (VX-950) | Generon/Adooq Bioscience | Cat# A10902-2 |  |
| chemical compound, drug | CsA-like CypI (JW115) | This paper |  | Generated in Prof. Dave Selwood’s lab (UCL), see Supplementary File 1. Synthesis of novel CypI. |
| chemical compound, drug | Depsin CypI (JW3-38) | This paper |  | Generated in Prof. Dave Selwood’s lab (UCL), see Supplementary File 1. Synthesis of novel CypI. |
| chemical compound, drug | CsA-Prtc1  (JW4-10) | This paper |  | Generated in Prof. Dave Selwood’s lab (UCL), see Supplementary File 1. Synthesis of novel CypI. |
